# Continuation of Long-Term Care for Cervical Dystonia at an Academic Movement Disorders Clinic

**DOI:** 10.3390/toxins5040776

**Published:** 2013-04-23

**Authors:** Chandler E. Gill, Neil D. Manus, Michael W. Pelster, Jason A. Cook, Wallace Title, Anna L. Molinari, David Charles

**Affiliations:** 1 Loyola University, Stritch School of Medicine, 2160 First Avenue South, Maywood, IL 60153, USA; E-Mail: cgill1@lumc.edu; 2 Vanderbilt University School of Medicine, 215 Light Hall, Nashville, TN 37232, USA; E-Mails: neil.manus@vanderbilt.edu (N.D.M.); michael.w.pelster@vanderbilt.edu (M.W.P.); 3 University of Minnesota Medical School, 420 Delaware Street Southeast, Minneapolis, MN 55455, USA; E-Mail: cook.jason.a@gmail.com; 4 Vanderbilt University Medical Center, Department of Neurology, 1161 21st Avenue South, MCN Suite A-1106, Nashville, TN 37232, USA; E-Mails: titlewj@gmail.com (W.T.); anna.l.molinari@vanderbilt.edu (A.L.M.)

**Keywords:** cervical dystonia, access to care, treatment, abobotulinumtoxinA, onabotulinumtoxinA, rimabotulinumtoxinB, botulinum neurotoxin

## Abstract

Patients with cervical dystonia (CD) receive much of their care at university based hospital outpatient clinics. This study aimed to describe the clinical characteristics and treatment experiences of patients who continued care at our university based movement disorders clinic, and to document the reasons for which a subset discontinued care. Seventy patients (77% female) were recruited from all patients at the clinic (*n* = 323). Most (93%) were treated with botulinum neurotoxin (BoNT) injection, and onabotulinumtoxinA was initially used in 97%. The average dose of onabotulinumtoxinA was 270.4 U (range 50–500) and the median number of injections was 14 (range: 1–39). Twenty one patients later received at least one cycle of rimabotulinumtoxinB (33%); of those, 10 switched back to onabotulinumtoxinA (48%). The initial rimabotulinumtoxinB dose averaged 11,996 units (range: 3000–25,000 over 1–18 injections). Twenty one patients (30%) discontinued care. Reasons cited included suboptimal response to BoNT therapy (62%), excessive cost (24%), excessive travel burden (10%), and side effects of BoNT therapy (10%). Most patients (76%) did not seek further care after leaving the clinic. Patients who terminated care received fewer treatment cycles (5.5 *vs*. 13.0, *p* = 0.020). There were no other identifiable differences between groups in gender, age, disease characteristics, toxin dose, or toxin formulation. These results indicate that a significant number of CD patients discontinue care due to addressable barriers to access, including cost and travel burden, and that when leaving specialty care, patients often discontinue treatment altogether. These data highlight the need for new initiatives to reduce out-of-pocket costs, as well as training for community physicians on neurotoxin injection in order to lessen the travel burden patients must accept in order to receive standard-of-care treatments.

## 1. Introduction

Patients with cervical dystonia (CD) experience involuntary contractions of the neck musculature that cause torticollis, laterocollis, retrocollis, and anterocollis. The resulting tremor, pain and abnormal postures are typically life-long. Chemodenervation is a standard of care, first line therapy for CD and involves temporary interruption of transmission at the neuromuscular junction with purified botulinum neurotoxins (BoNT), now available commercially as onabotulinumtoxinA (Botox^®^, Allergan, Irvine, CA, USA), abobotulinumtoxinA (Dysport^®^, Ipsen, Paris, France), incobotulinumtoxinA (Xeomin^®^, Merz, Frankfurt, Germany) and rimabotulinumtoxinB (Myobloc^®^, Solstice, Malvern, PA, USA). The muscle weakness produced by BoNT injection is uniquely focal, improving head position and pain without the sedation often associated with oral medications or the permanency of stereotactic surgical procedures. Effective administration of BoNT requires specialized training and detailed knowledge of the neck musculature and is often administered by neurologists with fellowship training in movement disorders. 

Despite symptomatic improvement with BoNT, CD patients often discontinue care. Occasionally, treatment becomes unnecessary due to disease remission; however, this occurs in only 5% of patients [1]. The reasons for which the great majority of CD patients discontinue treatment remain unknown. Previous retrospective analyses have been conducted [2,3], but have not directly answered this question; furthermore, the reasons behind discontinuation of care may differ between healthcare systems and geographic locations. The primary goal of this study was to describe the clinical characteristics and treatment experience of CD patients who have continued care at our university-affiliated movement disorders clinic, and to compare these characteristics to patients who discontinued care. The second goal was to determine the reasons for which CD patients discontinue care at academic centers. 

## 2. Results and Discussion

### 2.1. Results

#### 2.1.1. Patients

Seventy patients were randomly selected from the CD patient population at the clinic; they were 16 males and 54 females, resulting in a gender ratio of 1:3.4 (Table 1). Of the 67 patients who chose to report race, 65 were Caucasian (97.0%), one was African American (1.5%), and one was American Indian (1.5%). The average age of symptom onset was 45.0 ± 16.6 years (mean ± standard deviation), and patients delayed an average of 2.9 years before first seeking treatment. Most patients experienced a combination of dystonic movements. The most common movements were torticollis (83%) and laterocollis (50%) followed by retrocollis (11%) and anterocollis (7%). The most common neurological disorders present in the family history were essential tremor (17%), dystonia (7%), and Parkinson’s disease (4%). Most patients had been given at least one incorrect diagnosis prior to eventually receiving a diagnosis of CD; the most common was anxiety (9%) followed by psychogenic illness (7%) and compressed nerve (4%). Head or neck trauma within eight weeks of onset of CD symptoms was reported by 26% of patients.

**Table 1:** Characteristics of patients who continued and discontinued long-term care at an academic movement disorders clinic 1997–2006.

	All patients (*n* = 70)	Continued care (*n* = 49)	Discontinued care (*n* = 21)	*p*-Value
Gender	16 M:54 F	13 M:36 F	3 M: 18 F	0.216
Age of symptom onset	45.0 ± 16.6	44.3 ± 15.0	46.6 ± 18.4	0.617
Age at first treatment	47.9 ± 14.7	46.4 ± 14.4	51.2 ± 15.4	0.227
Proportion of patients with				
Torticollis	58 (83%)	42 (86%)	16 (76%)	0.264
Laterocollis	35 (50%)	24 (49%)	11 (52%)	1.000
Retrocollis	8 (11%)	6 (12%)	2 (10%)	1.000
Anterocollis	5 (7%)	5 (10%)	0 (0%)	0.314
Pain	55 (79%)	39 (80%)	16 (76%)	0.775
Tremor	42 (60%)	29 (59%)	13 (62%)	1.000
Headache	19 (27%)	12 (24%)	7 (33%)	0.561
Received at least one injection	65 (93%)	45 (92%)	20 (95%)	0.313
Received both BoNT-A and BoNT-B	21 (30%)	12 (25%)	9 (43%)	0.252
Number of injections (median, range)	11.5 (0–39)	13 (0–39)	5.5 (0–29)	0.020 *
Average starting dose of BoNT-A (units)	237.6 ± 85.7	230.8 ± 74.3	252.0 ± 107	0.436
Average dose of BoNT-A (units)	267.2 ± 81.4	269.9 ± 71.9	262.0 ± 101	0.744
Average dose of BoNT-B (units)	12,770 ± 5228	14,065 ± 5398	10,756 ± 4509	0.270

* indicates statistical significance.

#### 2.1.2. Treatments and Response

**Figure 1:**
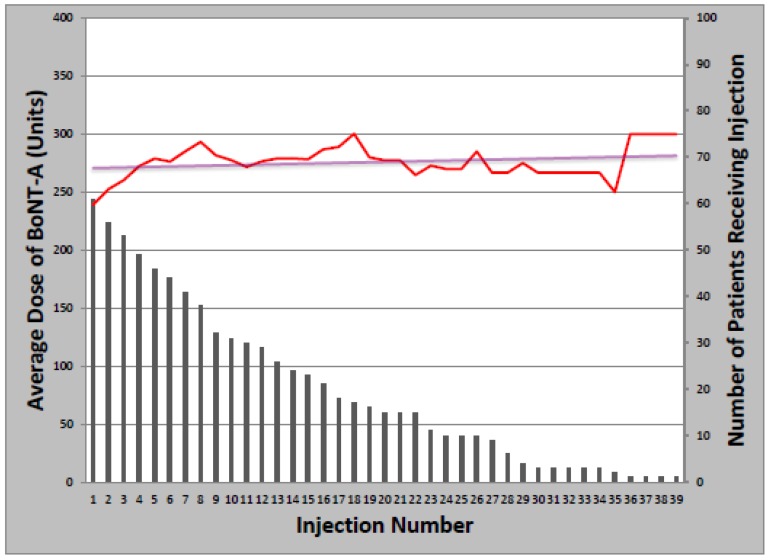
Change in onabotulinumtoxinA dose with multiple injections.

**Figure 2:**
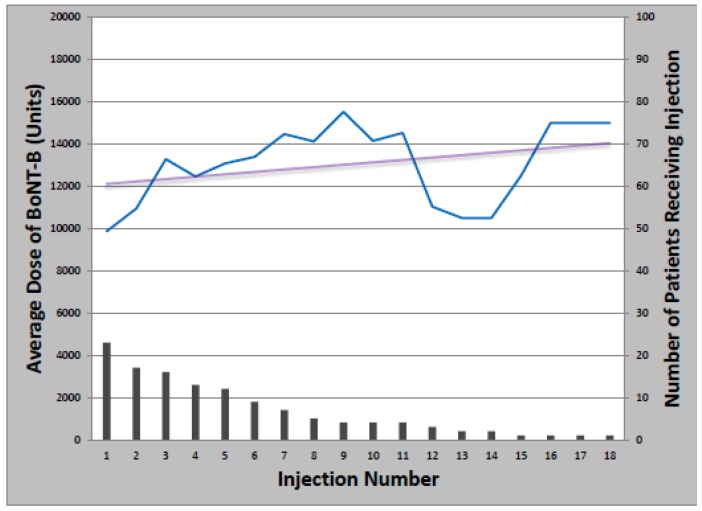
Change in rimabotulinumtoxinB dose with multiple injections.

The overwhelming majority of patients (93%) received at least one BoNT injection over the course of their treatment. Toxin formulations used during the study period were onabotulinumtoxinA, rimabotulinumtoxinB, and abobotulinumtoxinA. Treatment was initiated with onabotulinumtoxinA in 63 patients (97%); rimabotulinumtoxinB (1) and abobotulinumtoxinA (1) were injected first as part of clinical trials only. The mean starting dose of onabotulinumtoxinA was 237.6 ± 85.7 units, which remained relatively constant over successive injections, increasing an average of 1.1 units per year (Figure 1). Total dose ranged from 50 to 500 units and the median number of injections was 14 (Range: 1–39). Of patients initially treated with onabotulinumtoxinA, 21 later received at least one cycle of rimabotulinumtoxinB (33%); of those, 10 switched back to onabotulinumtoxinA (48%). Patients who received both onabotulinumtoxinA and rimabotulinumtoxinB received an average initial rimabotulinumtoxinB dose of 11,996 units which increased by 452 units per year. Average total dose per treatment cycle ranged from 3000 to 25,000 units over 1–18 injections (Figure 2). 

#### 2.1.3. Discontinuation of Treatment

Twenty-one patients (30%) had discontinued treatment at the time of the survey, most commonly for primary (4%) or secondary (11%) non-response to BoNT therapy (Table 2). Among the patients that experienced secondary non-response, three had documented toxin resistance by the “frontalis test” or by seropositivity for neutralizing antibodies. Of those no longer at the clinic, five sought care from other physicians; the remaining 16 (76%) were not receiving any form of treatment at the time of the telephone interview. 

**Table 2:** Reasons for discontinuation of care by cervical dystonia patients at a university-affiliated movement disorders clinic (*n* = 21).

Reason	Number of patients (% of discontinuees/% of total patients)
Secondary non-response to BoNT	8 (38.1/11.4)
Insufficient/No insurance	5 (23.8/7.1)
Primary non-response to BoNT	3 (14.3/4.3)
Long distance to clinic	2 (9.5/2.9)
Side effects experienced with BoNT	2 (9.5/2.9)
Other major illness	1 (4.8/1.4)
Spontaneous remission of CD	1 (4.8/1.4)

There were no differences between groups in gender, age of onset or treatment; nor did those who discontinued care display significantly different disease features than their successfully treated counterparts. Patients who terminated care received fewer treatment cycles (5.5 *vs*. 13.0, *p* = 0.020) but did not receive higher initial or average total doses of toxin (Table 1). 

### 2.2. Discussion

University based movement disorder clinics provide specialty services, such as BoNT injection, that are difficult to obtain in some communities. These services can lead patients to sacrifice convenience and, sometimes, expend significant personal resources traveling to and from university centers. Thus, it is not surprising that BoNT injection was a primary reason patients came to our clinic, with 93% of patients receiving at least one injection. Accordingly, the most common reasons for treatment discontinuation related to dissatisfaction with BoNT therapy. Both primary and secondary non-response were common and the rates were consistent with previous reports. Overall, 38% of patients who discontinued treatment did so because of secondary non-response (11% of the entire study population), which is close to the figure of 7.5% reported by Hsiung *et al.* [3]. Patients who continued care at the clinic received a moderate initial dose (231 units) which increased to an average of 270 units across all injection cycles. Those who discontinued care received an initial dose of 252 which increased only slightly to 262 units averaged across patients. The slightly higher initial dose could be attributed to a more complicated initial presentation as assessed by the treating physician. The smaller increase in total dose could represent the clinician’s reluctance to increase the dose due to bothersome side effects after the first injection, either due to the higher initial dose or inherent differences between patients. Interestingly, despite these disparate rates of dose increase, the overall doses received at our clinic were higher than those previously published for BoNT-A [2,3,4] but lower than those for BoNT-B [5]. Future analyses of our cohort should include more specific injection techniques including dose distribution among muscles and target localization using EMG guidance that may further impact patient satisfaction. For instance, several recent investigations have shown EMG guidance as a routine adjunct to clinical judgment enables greater reductions in muscle spasm using smaller toxin doses, leading to improved clinical outcomes [6,7]. 

Reasons unrelated to treatment efficacy were also commonly cited for treatment discontinuation; the most common was out-of-pocket expense, which was cited by significantly more patients in our study compared to previous reports. This discrepancy may illustrate unique considerations of our patient population. Specifically, the greater financial burden of care may indicate that our patients represent a lower socioeconomic demographic with lower rates of insurance coverage compared to patients in other areas. Inconvenience associated with travel also proved a major factor, in support of prior reports in which as many as 18% of cervical dystonia patients reported that poor accessibility to the clinic contributed to their decision to discontinue treatment [3]. Concerns over prohibitive costs and lengthy travel represent barriers to desirable outcomes that have practical solutions. To improve patient compliance, treatment must be affordable and conveniently accessible. Some foundations and BoNT manufacturers offer assistance programs to lessen the financial burden of treatment [8], but many patients or providers may not be aware of these programs, especially in rural areas such as the geographic area our clinic serves. Patient and provider outreach is thus an important factor in efforts to optimize access to care. Similarly, advocacy for better coverage of neurotoxin injection may reduce personal expenditures. Finally, efforts to reduce the travel burden associated with CD treatment could include education of community neurologists regarding BoNT injection techniques, thus eliminating the need to travel to university centers. 

There are several limitations to this study. First, our method of subject recruitment (telephoning patients during business hours) was different from previous studies that used clinic visits [3] and mail-in surveys [2] and may have led to the overestimation of certain demographic variables such as female gender (77% in this study). The generalizability is further limited by the small sample size. However, one advantage of this methodology is that by sampling a cross-section of clinic patients without minimum criteria for length of treatment, patient reports of response and side effects are less likely to suffer from selection bias than previous studies that included only participants who continued long-term care [3] and are therefore more likely to remain satisfied with care and treatment response.

## 3. Experimental Section

### 3.1. Study Patients

University IRB approval was obtained prior to study initiation and a waiver of written informed consent was granted. Inclusion criteria were (1) diagnosis of CD and (2) received care at the Vanderbilt University Movement Disorders Clinic at least once between 1997 and 2006. At the time of study initiation, there were six movement disorder specialists at the clinic, all of whom regularly administered BoNT. Treatment plans were formulated based on the clinical presentation and examination findings; electromyographic guidance was available throughout the study period. Because the study aimed to survey a random representative sample of clinic patients, there were no requirements for enrollment in terms of treatments received, toxin formulation, injection technique and method of guidance, or number of visits to the clinic. There were no exclusion criteria. All patients satisfying the inclusion criteria (*n* = 323) were contacted in random order until 70 patients completed the survey. 

### 3.2. Methods

Trained research assistants conducted a standardized medical record review to extract findings of interest from history and physical examination as described in the treating physician’s clinic note, including direction of involuntary movements, age at disease onset, age at which treatment was sought, treatments received prior to referral to the clinic, and misdiagnoses received prior to diagnosis of CD. For participants who received at least one injection of BoNT, toxin preparation and total dose were recorded for each administration. Finally, a standardized telephone interview was conducted to determine reasons for discontinuation of care at the clinic (if applicable) as well as any variable that could not be determined in the medical record review. 

### 3.3. Data and Analysis

Study data were collected and managed using Research Electronic Data Capture (REDCap) [9], a secure, web-based electronic data capture application hosted at Vanderbilt University. The Wilcoxon Rank-Sum Test was used to compare ages and neurotoxin doses between patients continuing *versus* discontinuing care. Categorical variables were compared using a two-tailed Fisher’s Exact Test. Significance level was *p* = 0.05 for all analyses. 

## 4. Conclusions

In this study, we attempted to clarify the reasons that lead patients to discontinue care at university based movement disorders clinics and discovered that while inadequate treatment response is often cited, a significant minority of patients leave due to addressable barriers in access to specialty care. This proved especially concerning because when leaving our clinic, patients often discontinued treatment altogether. Our data confirm the results of retrospective analyses of US metropolitan and non-US patient populations, and illuminate the need to: (1) reduce out-of-pocket cost, perhaps via advocacy for more complete insurance coverage and outreach efforts to increase awareness of patient assistance programs; and (2) provide training for community physicians on neurotoxin injection techniques, thus reducing the inconvenience and expense currently required for patients to receive the appropriate standard of care. 
